# The Potential of Brewer’s Spent Grain in the Circular Bioeconomy: State of the Art and Future Perspectives

**DOI:** 10.3389/fbioe.2022.870744

**Published:** 2022-06-17

**Authors:** Anđela Zeko-Pivač, Marina Tišma, Polona Žnidaršič-Plazl, Biljana Kulisic, George Sakellaris, Jian Hao, Mirela Planinić

**Affiliations:** ^1^ Faculty of Food Technology Osijek, Josip Juraj Strossmayer University of Osijek, Osijek, Croatia; ^2^ Faculty of Chemistry and Chemical Technology, University of Ljubljana, Ljubljana, Slovenia; ^3^ Energy Institute Hrvoje Požar, Zagreb, Croatia; ^4^ Czech Academy of Sciences (ASCR), Prague, Czechia; ^5^ Lab of Biorefinery, Shanghai Advanced Research Institute, Chinese Academy of Sciences, Pudong, China

**Keywords:** Brewer’s spent grain, bio-based products, circular bioeconomy, sustainability, biochemical transformation

## Abstract

Brewer’s spent grain (BSG) accounts for approximately 85% of the total mass of solid by-products in the brewing industry and represents an important secondary raw material of future biorefineries. Currently, the main application of BSG is limited to the feed and food industry. There is a strong need to develop sustainable pretreatment and fractionation processes to obtain BSG hydrolysates that enable efficient biotransformation into biofuels, biomaterials, or biochemicals. This paper aims to provide a comprehensive insight into the availability of BSG, chemical properties, and current and potential applications juxtaposed with the existing and emerging markets of the pyramid of bio-based products in the context of sustainable and circular bioeconomy. An economic evaluation of BSG for the production of highly valuable products is presented in the context of sustainable and circular bioeconomy targeting the market of Central and Eastern European countries (BIOEAST region).

## Graphical Abstract

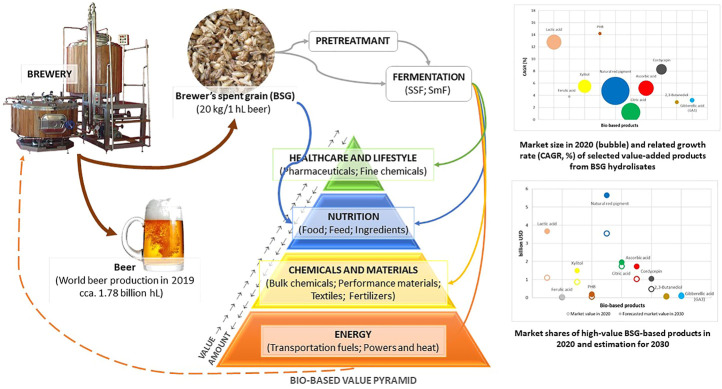

## Introduction

The circular and sustainable bioeconomy are gaining increasing attention as a means to address climate changes and defosillisation, to increase resource efficiency, and to create new opportunities for sustainable, long-term economic growth (Birner, 2018; European Commission 2018). In the search for innovative solutions to return wastes and by-products to the production cycle, the bioeconomy often relies on bioprocesses involving cells or their constituents. Biorefineries replace fossil carbon with renewable carbon from various lignocellulosic biomass resources to produce a range of bio-based products (Tišma et al., 2021a; Karp et al., 2021). The trend towards innovative biotechnological solutions for the valorization of lignocellulosic side-streams in the food and beverage industry is evident also from six projects funded by Bio-based Industries Joint Undertaking in 2020 (Bio-based Industries Joint Undertaking, 2020)^1^: VAMOS, HYPERBIOCOAT, IFERMENTER, VEHICLE, CAFIPLA, FIRST2RUN, and BIOSUPPACK, the latter considering also BSG.

Lignocellulosic materials are renewable raw materials derived from natural sources or bio-based chemical/biotechnological processes. They are mostly used directly or indirectly as feed or as a bioenergy source, but there is growing interest in using them as a substitute for fossil carbon in the production of various products including high-value chemicals and biomaterials (Tišma et al., 2021a). The main structural components of lignocellulosic materials are polymers, namely cellulose, hemicellulose, and lignin. The composition varies greatly depending on the type of raw material, harvesting and growth conditions, as well as handling and storage (Tišma et al., 2021a).

BSG is a lignocellulosic material that, along with hot trub and residual brewer’s yeast, accounts for the majority (approximately 85%) of solid by-products in the brewing industry (Lynch et al., 2016). In the beer production chain, the grain (mostly barley) is converted into malt in the malting process (steeping, germination and kilning), which takes place in the malting factory. Then, the malted barley (or other raw materials, alone or in combination, depending on the type of beer produced) goes through the mashing phase in the brewery, where milled malt and water are mixed and the hydrolytic enzymes responsible for breaking down the starch and proteins are activated. At the end of these processes, a mixture of undegraded and extracted ingredients in water is obtained. The aqueous solution containing the extract is called wort, while BSG is the solid phase that serves as a filter for the wort, which is further processed before being used as a medium for fermentation. The mass of wet BSG obtained per 1 hl of beer produced is about 20 kg (Gupta et al., 2010).

BSG is mainly used in feed and food production but the trend of converting BSG into products in the upper part of the bio-based value pyramid is evident from recent reviews (Lynch et al., 2016; Ikram et al., 2017; Abd El-Hack et al., 2019; Lao et al., 2020; Jaeger et al., 2021; Marcus and Fox, 2021; Puligundla and Mok, 2021). The establishment of a circular green process for BSG management is based on substantial and detailed experimental research of each process. Examples include the dehydration process of BSG, oil extraction, ethanol production and anaerobic digestion on a common basis, as a concept of an integrated multi-product biorefinery (Kavalopoulos et al., 2021).

This review aims to outline the availability of BSG with a focus on the EU and the 11 member states of The Central-Eastern European Initiative BIOEAST (2021),^2^. The chemical composition and current application of BSG are discussed, followed by the possibilities of biotransformation to achieve higher economic value. Generally, great emphasis has been placed on waste recovery through the development of high value-added products in order to provide new economic opportunities through the commercialization of products. The novelty of this paper is reflected in the current and forecasted assessment of market size of high-value products based on new BSG valorization research in the context of sustainable and circular bioeconomy.

## Systematic Review Protocol/Strategy

It is known that a detailed systematic literature search and strategy setting provides an explanation, hypothesis and planned order of the review paper. Protocol preparation ensures consistency, review, research integrity, and transparency of completed work (Moher et al., 2015). The systematic search strategy in this paper was undertaken following Systematic Review in Conservation and Environmental Management guidelines established by Pullin and Stewart (Pullin and Stewart, 2007) and at the beginning of a systematic search, a question-setting composed: a) subject, b) intervention, and c) research outcome was set. The guidelines of the model Preferred Reporting Items for Systematic Review and Meta-Analysis Protocols (PRISMA-P) established by Moher et al. (Moher et al., 2009) were also followed. The protocol of this systematic search is divided into three sections. First, a systematic literature search was conducted to collect all relevant data on the worldwide availability of BSG, followed by the chemical composition of BSG and its current use. Then, a systematic literature search was conducted to collect information on the potential use of BSG in the circular economy: a) the treatment of BSG with microorganisms or enzymes, b) the use of BSG as a substrate for the cultivation of microroganisms for enzyme production, c) the use of BSG as a substrate for the cultivation of microorganisms and for the production of high-value products. In the last section of the paper, the market size of high-value products was analyzed.

## Worldwide Beer Production

In 2020, the global annual beer production was estimated at 1.82 billion hL, with North and South America leading in production (615.28 million hL), followed by Asia (550.88 million hL), Europe (500.93 million hL), Africa (131.51 million hL) and Australia/Oceania (20.99 million hL) (Statista, 2021)^3^. Based on the global beer production quantities, about 36.4 million tons of BSG would be available worldwide. Table 1 presents global beer and BSG production by region.

**TABLE 1:** Worldwide beer and BSG production.

Production Category	Quantity of item produced by region
Worldwide beer production in 2020	
North and South America	615.28 billion hL
Asia	550.88 billion hL
Europe	500.93 billion hL
Africa	131.51 billion hL
Australia/Oceania	20.99 billion hL
Worldwide BSG production in 2020^a^	
North and South America	12.306 million tonnes
Asia	11.018 million tonnes
Europe	10.019 million tonnes
Africa	2.63 million tonnes
Australia/Oceania	0.42 million tonnes

a Assuming that BSG, is about 20 kg/hl brewed beer (Gupta et al., 2010), the amount of BSG, produced in relation to the amount of beer produced was calculated from data reference (Statista, 2021).

The Central-Eastern European Initiative BIOEAST supports transition to circular and sustainable bioeconomies comprising sustainable production and processing of residual biomass. BIOEAST member countries produce about 26% of the total EU27 beer production, meaning that BSG is an abundant resource to consider when creating a regional and national bioeconomical strategy (BIOEAST, 2021). Among the BIOEAST countries, the largest potential for BSG processing to higher added-value bio-based products is in Poland with annual BSG production of 816 kt, followed by Czech Republic (403 kt) and Romania (359 kt). Other member countries are Hungary (120 kt), Bulgaria (96 kt), Croatia (65 kt), Lithuania (63 kt), Slovakia (34 kt), Estonia (28 kt), Slovenia (19 kt) and Latvia (15 kt) in 2019. According to 2021 data from European Beer Association, there are currently around 11,000 active breweries in the EU producing around 400 million hL of beer per year. Calculated based on market movements of the last 5-year period, in 2030 the EU will produce about 425 million hL of beer and 8.5 million tonnes of BSG per year, which makes BSG an interesting biomass resource for the future biorefineries (The Brewers of Europe, 2019)^4^.

### Chemical Composition of BSG

The chemical composition of BSG depends on several factors, primarily on the type of barley (or other raw materials) used in malting, the time and technique of harvesting, the quality of the malt, the additives applied in the mashing stage, etc. (Lynch et al., 2016). For example, it has been shown that BSG produced from light malt has a higher concentration of phenolic compounds compared to dark malt obtained by malt roasting. Total amount of ferullic acid (FA) and *p*-coumaric acid (*p*-CA) was four fold higher in BSG obtained from light malt than in those from dark malt. The highest levels obtained for the light malt type were 1809.5 μg FA/g_DM_ and 686.6 μg *p*-CA/g_DM_, while those for the dark malt type were 404.7 μg FA/g_DM_ and 185.3 μg *p*-CA/g_DM_ (Birsan et al., 2019). During beer production, various additives such as gum arabic, propylene glycol alginate, polypeptides, zinc, iron, nickel and other elements are added that can improve foaming, cap hanging and foam stability. The addition of additives leads to hydrolysis of β-glucans, removal of oxalic acid, and polyphenols reduction, which also affects the final chemical composition of BSG (Li et al., 2010). In addition, the BSG storage process also causes changes in chemical composition. It was investigated that frozen samples had a higher content of protein and fat compared to lyophilized and oven-dried samples, but lower sugar content, especially arabinose (Johnson et al., 2010).

BSG is lignocellulosic material. The main components of BSG are hemicellulose, cellulose, lignin, proteins, and polysaccharides (Parchami et al., 2021). Among them, hemicellulose is the most abundant (19%–20%_DM_) (Tišma et al., 2018; Giacobbe et al., 2019) and in some cases reaches up to 41.3% (Assefa and Jabasingh, 2020), followed by cellulose (15.2%–28.7%_DM_) (Rojas-Chamorro et al., 2019; Assefa and Jabasingh, 2020). The average content of lignin is 11.41%, but it can vary from 3.35% (Castro and Colpini, 2021) to 21% (Giacobbe et al., 2019). The amount of arabinoxylans, the main components of hemicellulose, in BSG ranges from 2.67% (Bravi et al., 2021) to 21.9% (Lynch et al., 2021). β-glucans are present in an amount of 1% (Bravi et al., 2021). Total proteins range from 18.5% to 24.7% (Almeida et al., 2017; Nazzaro et al., 2020). The other presented components are lipids (8.4%) (Nazzaro et al., 2020), starch (5.3%) (Rojas-Chamorro et al., 2019) and ash (3.7%) (Sibhatu et al., 2021).

BSG is also comprised of phenolic compounds (Tišma et al., 2018), which are distributed among different parts of the barley kernel. Their concentration varies depending on the genotype of malt used, growing environment, and their interaction during production process (Rahman et al., 2021). According to Sajib et al., the most common phenolic compounds in BSG are ferulic acid (1,219.40 μg/g) and *p*-coumaric acid (488.51 μg/g). Smaller amounts of catechin,4-hydroxybenzoic acid, sinapic acid, syringic acid, protocatechuic acid and caffeic acid are also found (Birsan et al., 2019). The fatty acids found in BSG are palmitic acid (1.805 mg/g), stearic acid (0.596 mg/g), oleic acid (0.041 mg/g), linoleic acid (0.445 mg/g) (Tan et al., 2019). The amount of total amino acids in BSG is 0.859 mg/g, in the largest amount is proline (0.349 mg/g), then glutamic acid (0.340 (mg/g). In a smaller amount there are presence of aspartic acid, achenylalanine, serine, threonine, lysine and tyrosine (Tan et al., 2019). The most abundant sugars are glucose (37.06%), xylose (10.25%), arabinose (4.50%), mannose (1.18%) and galactose (0.24%) (Sajib et al., 2018). Various minerals found in ash are phosphorus (4,882.7 mg/kg), potassium (1,570.9 mg/kg), iron (210 mg/kg), calcium (81.60 mg/kg), zinc (67.2 mg/kg), manganese (34.3 mg/kg) (Almeida et al., 2017). BSG contains a significant amount of vitamins including vitamin B1 (25 mg/kg), vitamin B2 (25 mg/kg), vitamin B2 (9 mg/kg) and vitamin K (4.5 mg/kg) (Nagy and Diosi, 2021). Table 2 presents the chemical composition of BSG collected from literature, expressed as the mean value with indicated standard deviations.

**TABLE 2:** Estimated mean values of BSG primary components calculated from the literature data stated in the table. The values are expressed as a percentage of dry matter (%_DM_), or with an appropriate unit of measurement, with indicated standard deviations.

Compound [%_DM_]	Mean values	References
Hemicellulose	30.60 ± 9.78	Giacobbe et al. (2019), Tišma et al. (2018), Rojas-Chamorro et al. (2019), Sibhatu et al. (2021), Corchado-Lopo et al. (2021), Llimos et al. (2020), Assefa and Jabasingh (2020)
Cellulose	21.42 ± 4.81	Rojas-Chamorro et al. (2019), Tišma et al. (2018), Sibhatu et al. (2021), Llimos et al. (2020), Corchado-Lopo et al. (2021), Castro and Colpini (2021), Assefa and Jabasingh (2020)
Lignin	11.41 ± 6.76	Castro and Colpini (2021), Rojas-Chamorro et al. (2019), Llimos et al. (2020), Corchado-Lopo et al. (2021), Tišma et al. (2018), Sibhatu et al. (2021), Assefa and Jabasingh (2020), Giacobbe et al. (2019)
Proteins	20.93 ± 2.38	Castro and Colpini (2021), Almeida et al. (2017), Pathania et al. (2018), Rojas-Chamorro et al. (2019), Sibhatu et al. (2021), Nazzaro et al. (2020)
Lipids	8.52 ± 2.17	Castro and Colpini (2021), Almeida et al. (2017), Nazzaro et al. (2020), Naibaho and Korzeniowska (2021)
Starch	10.21 ± 10.97	Nazzaro et al. (2020), Giacobbe et al. (2019), Rojas-Chamorro et al. (2019)
Ash	3.68 ± 0.88	Rojas-Chamorro et al. (2019), Castro and Colpini (2021), Giacobbe et al. (2019), Almeida et al. (2017), Sibhatu et al. (2021), Pathania et al. (2018), Assefa and Jabasingh (2020), Nazzaro et al. (2020), Naibaho and Korzeniowska (2021)
Arabinoxylan	10.37 ± 10.17	Bravi et al. (2021), Lynch et al. (2021)
β-glucans	1 ± 0.00	Bravi et al. (2021)
Phenolic compounds [μg/gDM]		
Ferulic acid	1,144.53 ± 705.38	Sajib et al. (2018), Birsan et al. (2019)
*p*-Coumaric acid	453.47 ± 252.48	Sajib et al. (2018), Birsan et al. (2019)
Catechin	29.39 ± 47.64	Birsan et al. (2019), Almeida et al. (2017)
4-Hydroxybenzoic acid	14.89 ± 2.50	Birsan et al. (2019)
Sinapic acid	11.13 ± 4.95	Birsan et al. (2019)
Syringic acid	77.5 ± 44.16	Birsan et al. (2019), Almeida et al. (2017)
Protocatechuic acid	3.65 ± 0.26	Birsan et al. (2019)
Caffeic acid	0.28 ± 0.18	Birsan et al. (2019)
Amino acids [mg/g]		
Leucine	0.212 ± 0.140	Tan et al. (2019), Cooray and Chen, (2018)
Serine	0.020 ± 0.006	
Aspartic Acid	0.170 ± 0.206	
Threonine	0.036 ± 0.029	
Phenylalanine	0.089 ± 0.095	
Proline	1.128 ± 1.102	
Glutamic Acid	0.353 ± 0.069	
Lysine	0.056 ± 0.062	
Tyrosine	0.053 ± 0.066	
Fatty acids [mg/g]		
Palmitic acid	1.029 ± 1.099	Tan et al. (2019), Almeida et al. (2017)
Stearic acid	0.303 ± 0.415	
Oleic acid	0.072 ± 0.044	
Linoleic acid	0.506 ± 0.087	
Sugars [%]		
Glucose	23.06 ± 13.38	Sajib et al. (2018), Meneses et al. (2013), Denstadli et al. (2010)
Xylose	12.96 ± 2.44	Sajib et al. (2018), Meneses et al. (2013), Denstadli et al. (2010)
Arabinose	5.95 ± 1.62	Sajib et al. (2018), Meneses et al. (2013), Denstadli et al. (2010)
Mannose	0.94 ± 0.34	Sajib et al. (2018), Denstadli et al. (2010)
Galactose	0.77 ± 0.75	Sajib et al. (2018), Denstadli et al. (2010)
Minerals [mg/kg]		
Phosphorus	5,441.35 ± 790.05	Almeida et al. (2017), Meneses et al. (2013)
Potassium	1,085.45 ± 686.53	
Iron	182.45 ± 38.97	
Calcium	1,840.8 ± 2,487.89	
Zinc	74.65 ± 10.54	
Manganese	37.6 ± 4.67	
Vitamins [mg/kg]		
Vitamin B1	25	Nagy and Diosi (2021)
Vitamin B2	25	
Vitamin B6	9	
Vitamin K	4.50	

Conventional techniques for determining the chemical composition of BSG are complex and time consuming, so novel techniques that are both fast and precise are requested. A promising, cost-effective, and widely used technique for rapid determination of chemical composition can be near-infrared spectroscopy (NIR). NIR is used to characterize the chemical composition by providing qualitative and quantitative information on complex samples, in BSG, the presence of cellulose, hemicellulose and lignin was investigated by this method (Amirvaresi et al., 2020; Castro and Colpini, 2021). Thermogravimetry (TG) is a modern and fast technique that provides accurate and fast measurements. TG is used to characterize the composition and moisture content of lignocellulosic material by continuous monitoring a decrease or increase in mass as a function of temperature applied (Rego et al., 2019). The amount and distribution of cellulose, hemicellulose, and lignin to submicroscopic level can be elucidated using the Raman scattering microscopy technique. A potential problem with spectroscopic techniques is the accurate determination of the peaks associated with the component of interest due to the intricate nature of the basic spectrum (Krasznai et al., 2017).

### The Most Common Use of BSG

At present, BSG is used for various purposes in wet form, directly after filtration, or in dried form after drying. Traditionally it is used in feed and food production, but recently BSG also plays a role in bioenergy production and waste management. The cost of transportation (especially of BSG in wet form), the cost of drying, and need for pretreatment process to reduce its recalcitrance, are some of the barrier to wider application (Pabbathi et al., 2022).

Its use in animal feed is related to its high fiber and protein content. However, the high fiber content has positive effects on cows and negative effects on poultry. Feeding cows with BSG improves digestibility and has a positive effect on milk production efficiency and profitability, but may also reduce dry matter intake, body weight, protein and milk fat (Faccenda et al., 2017). Dried BSG contains the nutrients needed in poultry feed formulation, but its use may be limited due to high fiber content, resulting in reduced digestibility (Abd El-Hack et al., 2019). BSG is also used in pig and lamb feeding and, contributes to weight gain and meat quality (Amoah et al., 2017; Radzik-Rant et al., 2018).

Recent studies show that BSG is a potential source of prebiotics that optimize the balance and activity of microbes in the gut. When domestic animals ingest arabinoxylans and β-glucans from BSG, the activity of beneficial bacteria, especially *Bifidobacterium, Enterococcus and Lactobacillus*, is stimulated. In addition, increased degradation of dietary fiber accelerates the production of short-chain fatty acids, which are the primary energy source for anaerobic microbes. With appropriate considerations and a BSG strategy as a dietary supplement, the overall health and breeding of domestic animals can be improved (Lao et al., 2020).

In the food industry, BSG is used for the production of bread (Neylon et al., 2021), cookies (Farcas et al., 2021), muffins (Combest et al., 2020), pasta (Schettino et al., 2021), cereal bars (Stelick et al., 2021), chips (Garrett et al., 2021) and yogurt (Naibaho et al., 2022). The increased content of dietary fiber helps in the elimination of cholesterol and fats and improves the symptoms of ulcerative colitis. Moreover, the presence of phenolic compounds, which are considered as natural antioxidants, is associated with the prevention of chronic, cardiovascular, and neurogenerative diseases, certain cancers, and diabetes (Ikram et al., 2017). Additionally, BSG is a suitable medium for the growth of various fungi, bacteria and other microorganisms due to its chemical composition, particle size, and water retention capacity (Xiros and Christakopoulos, 2012; Malomo et al., 2013).

BSG can also be used as a vermicompost/soil improver, after bioconversion with *Eisenia fetida* in combination with microorganisms. The suitability of BSG as a substrate for the growth of these worms is demonstrated by the results of reduced total organic carbon, increased total nitrogen and total humus matter, which induces enhanced mineralization and stabilization (Saba et al., 2019).

## The Potential Use of BSG in Circular Bioeconomy

The concept of circular bioeconomy is mainly aimed at the recovery of all products from the resources without generating waste. Lignocellulosic biorefineries are enablers of circular bioeconomy, while through integrated biorefinery processes, multiple end products could be produced. However, to meet the sustainability requirements, development and implementation of sustainable technologies in conversion of BSG and other lignocellulosic materials into bio-basaed products are urgently needed (Sganzerla et al., 2021a). Sganzerla et al. (2021b) made techno-economic assessment of bioenergy and fertilizer production by anaerobic digestion of BSG with simulations performed by integrating the production of biomethane, electricity, thermal energy, and fertilizer, which is one of examples of application of brewer’s spent grains following biorefinery concept.

### Biochemical Transformation of BSG for Improved Applicability in Production and Environmental Processes

As mentioned above, the current use of BSG in wet or dry form shows some disadvantages in certain applications. Therefore, considerable efforts are being made to improve the BSG applicability through various pretreatment processes. Supplementary Table S1 provides an overview of the methods used to treat BSG for its further use in energy production, waste management, biofertilizer, feed and food production.

To improve the quality of BSG for use as feed, BSG can be treated with enzymes (Denstadli et al., 2010) or microorganisms. Solid-state fermentation (SSF) technology is a promising technology for the treatment of lignocellulosic materials for various applications (Tišma et al., 2021b). Xylanase pretreatment BSG for chicken feed reduced the reduced the concentration of polymeric arabinose and xylose by 15%–30% (Denstadli et al., 2010), and the inclusion of BSG treated with SSF by *Aspergillus ibericus* in fish diet increased dry matter digestibility and energy (Fernandes et al., 2021). SSF was used for BSG treatment for the production of food, wherein the properties of whole-wheat bread with the addition of fermented BSG by *Aspergillus awamory* increase the amount of ferulic acid by 198% (Dos Santos Costa et al., 2020). SSF with *Bacillus subtilis* resulted in improved nutritional composition of BSG. There was an increase in total amino acids by 2 fold, 1.7 fold of unsaturated fatty acids and 5.8 fold of antioxidants compared to unfermented BSG (Tan et al., 2019). In particular, the bioconversion of BSG *via* SSF improves the nutritional profile and indicates the potential use of BSG in a diet enriched with proteins and bioactive compounds. Thus, SSF by *Pleurotus ostreatus* resulted in an increase in the proportion of protein and 1,3/1,6-β-glucans in fermented BSG (Eliopoulos et al., 2021). Also, the implementation of SSF by *Rhizopus sp.* led to an 11 fold increase in total polyphenolic compounds and enrichment of BSG with amino acids (Ibarruri et al., 2019). BSG has proven to be a good substrate for the production of biocontrol fertilizer by fungal SSF (Qiu et al., 2019) and vermicompost enriched with bacteria and fungi (Bianco et al., 2022).

However, most studies on BSG pretreatment are devoted to its use for bioenergy production. One study considers silver nitrate pretreatment of BSG prior to pyrolysis (Ashman et al., 2020), while the majority of studies is dedicated to biogas (Panjičko et al., 2017; Dudek et al., 2019), bioethanol (Caetano et al., 2013), or biobutanol (Giacobbe et al., 2019) production. A two-stage biogas production process from BSG as a mono-substrate was developed, where methanogenesis was performed in a granular biomass reactor, while microbiological hydrolysis and acidogenesis were performed in a solid-state anaerobic digestion reactor. After adaptation of the microbial community, the process exhibited long-term stable operation and showed a high degradation efficiency and biogas/methane production capacity (Panjičko et al., 2017). Improvement of yields and increase of biogas production by anaerobic digestion of BSG can be achieved by adding biochar produced by the process of torefication, also from BSG. The maximum biogas production without the addition of biochar was 92.3 dm^3^/kg of dry organic matter (DOM), while it increased to 122 dm^3^/kg_DOM_ with the addition of 5% biochar. The addition of a high percentage of biochar (20%–50%) can inhibit biogas production (Dudek et al., 2019). In the work of Ortiz et al. (2019) it is shown that by gasification of BSG it is possible to achieve a net economic saving of 22%, if the produced gas would be used for heat production in breweries, with the advantage of minimizing BSG as waste.

The process of bioethanol production typically involves pretreatment of lignocellulosic material to enhance carbohydrate hydrolysis, further fermentation of simple sugars into ethanol, and final distillation for product recovery (Caetano et al., 2013). Widely used pretreatment techniques comprise acid hydrolysis, alkaline wet oxidation and steam explosion, which involve high temperatures and pressures and often lead to the formation of compounds that negatively affect the further fermentation of sugars into ethanol. In contrast, enzymatic hydrolysis, which is carried out under mild conditions, does not generate hazardous by-products (Heredia-Olea et al., 2015). For example, application of two laccase preparations from *Pleurotus ostreatus* on milled BSG resulted in up to 94% of phenols reduction. Moreover, the formation of other inhibitory compounds was avoided, so further fermentation to acetone-butanol-ethanol using *Clostridium acetobutilycum* was enhanced (Giacobbe et al., 2019). The high yield of biobutanol obtained in this study is noteworthy because butanol has a higher energy value, lower vapor pressure, lower corrosivity, and lower tendency to mix with water compared to ethanol, and can be transported through existing pipelines (Bellido et al., 2014). The use of hydrolysate from various lignocellulosic materials has been investigated a lot, suggesting that there is a need for exploring the use of BSG for isobutanol production. An engineered *Escherichia coli* strain yielded 3.7 g/L of biobutanol when grown on cedar hydrolyzate (Akita et al., 2015). This microorganism was also used by Minty et al. (2013) on corn stover, without costly nutrient supplementation, 1.88 g/L of isobutanol was produced. A synthetic fungal-bacterial consortia was developed, in which the fungus secretes cellulase enzymes to hydrolyze lignocellulosic material, and *E. coli* metabolizes soluble saccharides into isobutanol. An isobutanol producing strain that use *Clostridium thermocellum* as the host produced 5.4 g/L of isobutanol from cellulose (Lin et al., 2015).

In waste management, BSG can be used as a low-cost adsorbent to remove heavy metals such as silver and iron (Li et al., 2010; Izinyon et al., 2016), or to remove dyes such as Congo Red, Methylene Blue and Malachite Green dyes in the wastewater system (Kezerle et al., 2018; Chanzu et al., 2019). The modification of BSG is beneficial for the removal of silver from aqueous solutions. Various agents such as oxidizing agents, mineral and organic solutions, bases and acids have been used to improve the adsorption capacity of BSG (Li et al., 2010).

In an effort to reduce the production of synthetic materials and increase plastic recycling, BSG is a potential raw material for biofilm production. Biofilms used in food products and medicines must have thermal stability which has been successfully achieved with the arabinoxylan-rich fraction from BSG (Jaguey–Hernández et al., 2022). Also, due to the rich protein profile of BSG, Proaño et al. (2020) have used protein dispersion from BSG in the production of biofilms with potential application in active food packaging. Protein films showed balanced mechanical properties, water resistance and antioxidant capacity.

### BSG as a Resource for the Production of Enzymes and Other Value-Added Products

Biocatalysis and continuous processing were identified as crucial enabling technologies for the development of cost-efficient manufacturing with high-quality products and low waste generation, following the 12 principles of green chemistry (Žnidaršič-Plazl, 2021a; Žnidaršič-Plazl, 2021b). Enzymes are environmentally friendly catalysts that operate under mild conditions with high regio-, stereo-and reaction selectivity, making them key tools for single-step biotransformations up to total chemo- or multi-enzymatic syntheses (Wohlgemuth, 2021). Enzymes can be produced by submerged fermentation (SmF) or SSF, the latter being more suitable when the substrate for microbial growth and enzyme production is lignocellulose. The advantages and disadvantages of both fermentation techniques were described in recent review papers (Tišma et al., 2021b; Leite et el., 2021).

The possibilities of using BSG as a substrate for cultivation of various microorganisms in SSF to produce a variety of enzymes are shown in Table 3. Information about the microorganism, the type of cultivation and the enzyme activities obtained is indicated. The use of BSG as fermentation medium component typically results in high xylanase activities, which is to be expected considering that hemicellulose represents the largest portion of all polymers in BSG. In addition, many other enzymatic activities are involved in the complex degradation of lignocellulosic material (Šelo et al., 2021; Chilakamarry et al., 2022) e.g., various glucanases, cellobiohydrolases, esterases and pectinases.

**TABLE 3:** BSG as a substrate for the production of different enzymes by different microorganisms in SSF or SmF conditions.

Enzyme	Microorganism	Enzyme activity^a^	Literature
SSF Cultivation Technique			
Xylanase	*Mucor sp.*	S.A. = 67 U/g	Hassan et al. (2020)
Pectinase		S.A. = 137 U/g	
Xylanase	*Penicillium janczewskii*	S.A. = 370 U/g	Terrasan and Carmona (2015)
α-l-arabinofuranosidase		S.A. = 0.668 U/g	
β-xylosidase		S.A. = 285 U/g	
Xylanase	*Penicillium brasilianum*	S.A. = 709 U/g	Panagiotou et al. (2006)
α-l-arabinofuranosidase		S.A. = 3.57 U/g	
Feruloyl esterase		S.A. = 1.54 U/g	
Xylanase	*Fusarium oxysporum*	S.A. = 1090 U/g	Xiros et al. (2008a)
α-l-arabinofuranosidase		S.A. = 2.4 U/g	
Feruloyl esterase		S.A. = 0.36 U/g	
Endoglucanase		S.A. = 75 U/g	
Cellobiohydrolase		S.A. = 2.7 U/g	
Acetyl esterase		S.A. = 2.3 U/g	
β -D-glucosidase		S.A. = 1.3 U/g	
β -D-xylosidase		S.A. = 0.7 U/g	
Xylanase	*Neurospora crassa*	S.A. = 1073 U/g	Xiros et al. (2008b)
α-l-arabinofuranosidase		S.A. = 3.1 U/g	
Feruloyl esterase		S.A. = 0.52 U/g	
Endoglucanase		S.A. = 56 U/g	
Cellobiohydrolase		S.A. = 4.2 U/g	
Acetyl esterase		S.A. = 5.7 U/g	
β-glucosidase		S.A. = 1.6 U/g	
Xylanase	*Aspergillus niger*	1,400.80 U/g_DM_	Moran-Aguilar et al. (2021)
Cellulase		6.23 U/g_DM_	
Dextranase	*Penicillium aculeatum*	75.5 U/g_DM_	Batista et al. (2021)
Xylanase	*Aspergillus brasiliensis*	3,152.39 U/g_DM_	Outeiriño et al. (2019)
Laccase	*Trametes versicolor*	S.A. = 13,506.2 U/g	Dhillon et al. (2012)
		V.A. = 560 U/L	Tišma et al. (2018)
SmF Cultivation Technique			
Xylanase	*Humicola grisea* var. *thermoidea*	V.A. = 16.90 U/mL	Mandalari et al. (2008)
	*Talaromyces stipitatus*	V.A. = 2.33 U/mL	
Feruloyl esterase	*Humicola grisea* var. *thermoidea*	V.A. = 0.47 U/mL	Mandalari et al. (2008)
	*Talaromyces stipitatus*	V.A. = 0.14 U/mL	
α-Amylase	*Bacillus sp. KR-8104*	V.A. = 23.55 U/mL	Hashemi et al. (2011)
	*Bacillus stearothermophilus*	V.A. = 198.09 U/mL	Ravindran et al. (2019)

a S.A., specific activity; V.A., volume activity.

BSG is considered as a cheap and promising raw material for the production of high-value products (Xiros and Christakopoulos, 2012). The studies on the use of BSG for the production of lactic acid, ferulic acid, *p*-coumaric acid, xylitol, poly-3-hidroxybutyrate, natural red pigment, 2,3-butanediol, gibberellic acid, citric acid, ascorbic acid and cordycepin are summarized in Table 4. Table 4 provides information of pretreatment used to produce BSG hydrolyzate, including the techniques to concentrate simple sugars, and the data on the fermentation conditions comprising the microorganisms used to produce the product of interest together with the yield obtained.

**TABLE 4:** Production of value-added products from BSG hydrolysates.

Pretreatment methods^a^	Intermediate product(s) obtained after pretreatment	Fermentation type/medium/conditions	Microorganism(s)	Product	Yield level	Literature
P1: Acid hydrolysis (1.25% H_2_SO_4_; *T* = 120°C; *t* = 17 min)	BSG hydrolysate (50 g/L glucose)	SmF/BSG hydrolysate +5 M NaOH +1 g/L cell concentration/*V* = 25 ml; *T* = 37°C; *t* = 48 h	*Lactobacillus delbrueckii* UFV H2B20	Lactic acid	0.73 g lactic acid/g glucose consumed	Mussatto et al. (2007b)
P2: Alkali hydrolysis (2% NaOH; *T* = 120°C; *t* = 90 min)						
P3: Enzymatic hydrolysis (Cellulast complex; 45 FPU/g; 100 rpm; *t* = 96 h)						
P1: Acid hydrolysis (1.25% H_2_SO_4_; *T* = 120°C; *t* = 17 min)	BSG hydrolysate (75 g/L glucose)	SmF/BSG hydrolysate +5 M NaOH +10% cell suspension + yeast extract (or without)/*V* = 250 ml; *T* = 37°C; *t* = 96 h; 60 g	*Lactobacillus acidophilus* ATCC 43121	Lactic acid	0.48 g lactic acid/g glucose consumed from BSG hydrolysate (without add. of yeast extract)	Liguori et al. (2015)
P2: Alkali hydrolysis (2% NaOH; *T* = 120°C; *t* = 90 min)						
P3: Enzymatic hydrolysis (2.24% cellulase and 1% β-glucosidase; *T* = 45°C, 120 rpm; *t* = 72 h)					0.60 g lactic acid/g glucose consumed from BSG hydrolysate (with add. of yeast extract)	
Acid hydrolysis (1.5 M H_2_SO_4_; *T* = 130°C; *t* = 30 min)	BSG hydrolysate (39.85% glucose)	SmF/Culture media and BSG hydrolysate (1:10)/*T* = 35°C; *t* = 72 h; 200 rpm	*Lactobacillus plantarum* ATCC 8014	Lactic acid	27.78%	Assefa and Jabasingh (2020)
P1: Acid hydrolysis (72% H_2_SO_4_; *T* = 120°C; *t* = 17 min)	Lignin (solubilized 60 and 90%)	-	-	Ferulic acid, p-coumaric acid	9.65 mg ferulic acid/g of solubilized lignin and 9.22 mg p-coumaric acid/g of solubilized lignin	Mussatto et al. (2007a)
P2: Alkali hydrolysis (2% NaOH; *T* = 120°C; *t* = 90 min)						
Acid hydrolysis (H_2_SO_4_, *T* = 120°C; *t* = 17 min)	BSG hydrolysate (21.88 g/L xylose)	SmF/BSG hydrolysate +1 g/L cell concentration/*V* = 250 ml; *T* = 30°C; *t* = 24 h	*Candida guilliermondii* FTI 20 037	Xylitol	0.70 g xylitol/g xylose consumed	Mussatto and Roberto (2005)
Hydrothermal pretreatment (BSG + distilled water; *T* = 160°C; *t* = 20 min)	Hemicellulose liquor (20 g/L xylose)	SmF/BSG liquor + cell suspension/*V* = 2 ml; *T* = 30°C; 100 rpm; *t* = 24 h intervals	*Pachysolen tannophilus*	Xylitol	0.47 g xylitol/g xylose consumed	da Silva et al. (2019)
Enzymatic hydrolysis (dried BSG + enzymatic extract obtained after the SSF with *A. niger* + enzymatic cocktail Viscozyme L (1%); *t* = 48 h)	BSG hydrolysate (0.56 g of reducing sugar/gDM)	SmF/BSG hydrolysate +5% cell/*V* = 100 ml; *T* = 30°C; 120 rpm; *t* = 48 h (*C. necator*); *t* = 72 h; (*B*. *cepacia*)	*Burkholderia cepacia*	Poly-3-hidroxybutyrate (PHB)	7 mg PHB/gDM	Llimós et al. (2020)
			*Cupriavidus necator*		9 mg PHB/gDM	
P1: Acid hydrolysis (1–6% H_2_SO_4_, *T* = 120°C, *t* = 15 min)	Glucose, arabinose, xylose + added monosodium glutamate (8 g/L)	SmF/BSG hydrolysate +2% of spore solution + MSG, KH_2_PO_4_, K_2_HPO_4_, MgSO_4_·7H_2_O, CaCl_2_, and ZnSO_4_·7H_2_O/*V* = 50 ml; *T* = 30°C; *t* = 7 days; 350 rpm	*Monascus purpureus* CMU001	Natural red pigment	22.25 UA500	Silbir and Göksungur (2019)
P2: Detoxification (Ca(OH)_2_, *T* = 55°C, *t* = 1 h)						
P: Acid hydrolysis (1.5% H_2_SO_4_, *T* = 121°C, *t* = 20 min)	Glucose, xylose and arabinose (0.44 g/g total solids)	Acidogenic fermentation/BSG + anaerobic granular sludge/*V* = 1.75 L; *T* = 37°C; *t* = 72 h; 1,500 rpm	-	Volatile fatty acids	16.89 g COD/L	Castilla-Archilla et al. (2020)
P1: Microwave-assisted alkali hydrolysis (0.5% NaOH; microwave radiation 400 W; *t* = 60 s)	BSG hydrolysate (0.25 g glucose/g biomass)	SmF/BSG hydrolysate +2% of inoculum + (NH_4_)_2_HPO_4_, (NH_4_)_2_SO_4_, KOH, EDTA, MgSO_4_·7H_2_O, FeSO_4_⋅7H_2_O, CaCl_2_⋅6H_2_O, MnSO_4_⋅H_2_O and ZnSO_4_·7H_2_O/*V* = 100 ml; *T* = 30°C; 180 rpm	*Enterobacter ludwigii*	2,3-Butanediol	0.43 g 2,3-Butanediol/g glucose consumed from BSG hydrolysate	Amraoui et al. (2022)
P2: Enzymatic hydrolysis (cellulase 100 U/mL; *T* = 50°C, 150 rpm; *t* = 96 h)						
NP	Protein and carbon sources	SSF/BSG + inoculum + glucose, FeSO_4_⋅7H_2_O, MgSO_4_, MnSO_4_⋅H_2_O and ZnSO_4_·7H_2_O/*V* = 500 ml; *T* = 28°C, *t* = 96 h	*Fusarium fujikuroi*	Gibberellic acid (GA3)	0.82 g GA3/kg BSG	da Silva et al. (2021)
NP	Carbon source (sugars, CO_2_, biomass)	SmF/BSG + peptone, yeast extracts, KH_2_PO_4_, (NH_4_)2SO_4_, MgSO_4_·H_2_O+ methanol +0.1% of spore suspension/conival flask; *T* = 30°C; *t* = 14 days	*Aspergilus nige*r	Citric acid	0.512% mass per volume of citric acid	Femi-Ola and Atere (2012)
			*Saccharomyces cerevisiae*		0.312% mass per volume of citric acid	
NP	Carbon source (sugars)	SSF/BSG + peptone, yeast extract, KH_2_PO_4_, (NH_4_)_2_SO_4_, MgSO_4_·H_2_O/*V* = 150 ml	*Aspergilus niger* *strain*	Citric acid	0.23% mass per volume of citric acid	Pathania et al. (2018)
NP	Sugars (D-glucose)	SmF/BSG + D-glucose, l-galactose, yeast extract, peptone, monosodium glutamate/*T* = 40°C; *t* = 96 h; 100 rpm	*Aspergillus flavus*	Ascorbic acid	7.25 g/L	Banjo et al. (2018)
			*Aspergillus tamarii*		6.25 g/L	
NP	Ergosterol	SSF/50% BSG + rye, 5 ml liquid inoculum/*V* = 720 ml; *t* = 24 h; under cool white fluorescent light	*Cordyceps militaris*	Cordycepin	10.42 mg cordycepin/g substrate	Gregori (2014)

a P, pretreatment; P1, 1st step of pretreatment; P2, 2nd step of pretreatment; P3, 3rd step of pretreatment; NP, no pretreatmet.

The widespread use of lactic acid in the food, pharmaceutical, chemical, and textile industries requires industrial manufacturing on a ton scale. Its production from bio-based materials has increased significantly in the last decade due to the increasing use of (poly) lactic acid, a bio-based and biodegradable biopolymer used in many disposable packaging (Datta and Henry, 2006). Pretreatment is required to remove the lignin barrier from BSG, followed by saccharification of polysaccharides with a cellulolytic enzyme cocktail, and fermentation of the resulting sugars using microorganisms (Liguori et al., 2015). Citric acid is an important organic acid produced in tons as it is widely used in food industry and household. It can be produced from BSG by SmF using *Aspergillus niger* (Pathania et al., 2018). da Silva et al. (2021) published that BSG can be used as a growth medium for the cultivation of *Fusarium fujikuroi* to produce gibberellic acid (GA3), which has a promising application in the agroindustrial sector as it is related to plant growth.

The most common phenolic acids that can be isolated from lignin of BSG are ferulic and *p*-coumaric acid. Since these phenolic acids are precursors in the biocatalytic production of aromatic natural value-added compounds, BSG can be used as a source of these compounds (Mussatto et al., 2007a; Mussatto et al., 2007b). Xylitol is a functional sweetener that is usually produced chemically, but can also be produced from xylose-rich hydrolysates that can be obtained by lignocellulose degradation. BSG is a potential and cost-effective raw material for xylitol production without the need to add nutrients to the medium and detoxify the hydrolysate (Puligundla and Mok, 2021). An innovative approach to reducing the environmental impact of BSG processing in xylitol, ethanol, and polyhydroxybutyrate production in biorefineries using a heat integration strategy suggested that the overall production costs could be reduced by 43% (Dávila et al., 2016).

BSG also occupies a place in pharmaceutical use. It was used as a culture medium for *Cordyceps militaris* for the production of cordycepin (3′-deoxyadenosine), which has anti-tumor, anti-metastatic, anti-bacterial, anti-proliferative and insecticidal action (Gregori, 2014). 2,3-butanediol is a microbially synthesized metabolite with versatile use in the food, chemical and pharmaceutical industries with great market potential. The high cost of the substrate in the conventional production of this compound, gives preference to renewable raw materials such as BSG (Amraoui et al., 2022). A natural red pigment was also produced during SmF with BSG hydrolysate, where sodium glutamate was used as an additional nitrogen source that also stimulate the production of the pigment by *Monascus purpureus.* Maximum red pigment production of 22.25 UA500 was achieved at pH 6.5, 350 rpm shake speed, 50 ml volume and inoculation ratio 2% (v/v) (Silbir and Göksungur, 2019).

Among other alternatives associated with the use of BSG, it is worth mentioning the production of fatty acids (Castilla-Archilla et al., 2020) and the production of biodegradable plastics (e.g. polyhydroxyalkanoates, PHA) (Llimós et al., 2020), leading to fulfilment of the aspirations for a sustainable manufacturing and circular bioeconomy.

### Existing Market Size and Growth Rate of Selected Value-Added Products From BSG

**FIGURE 1:**
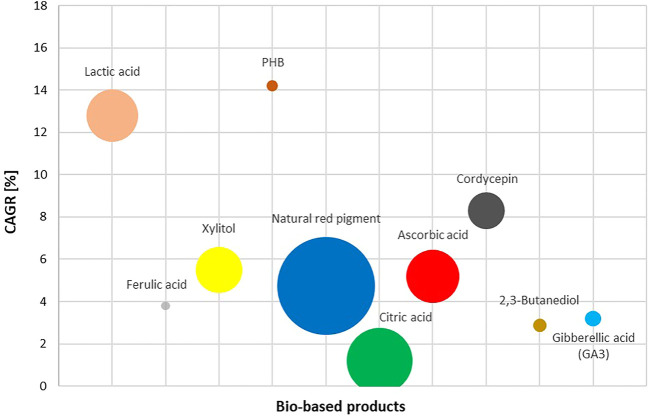
Market size and related growth rate (CAGR) of selected value-added products from BSG hydrolisates (PHB, poly-3-hydroxybutyrate (as a part of the PHA group); Natural red pigment as a part of natural pigment group). The size of the bubble represents the relative market size in 2020.

**FIGURE 2:**
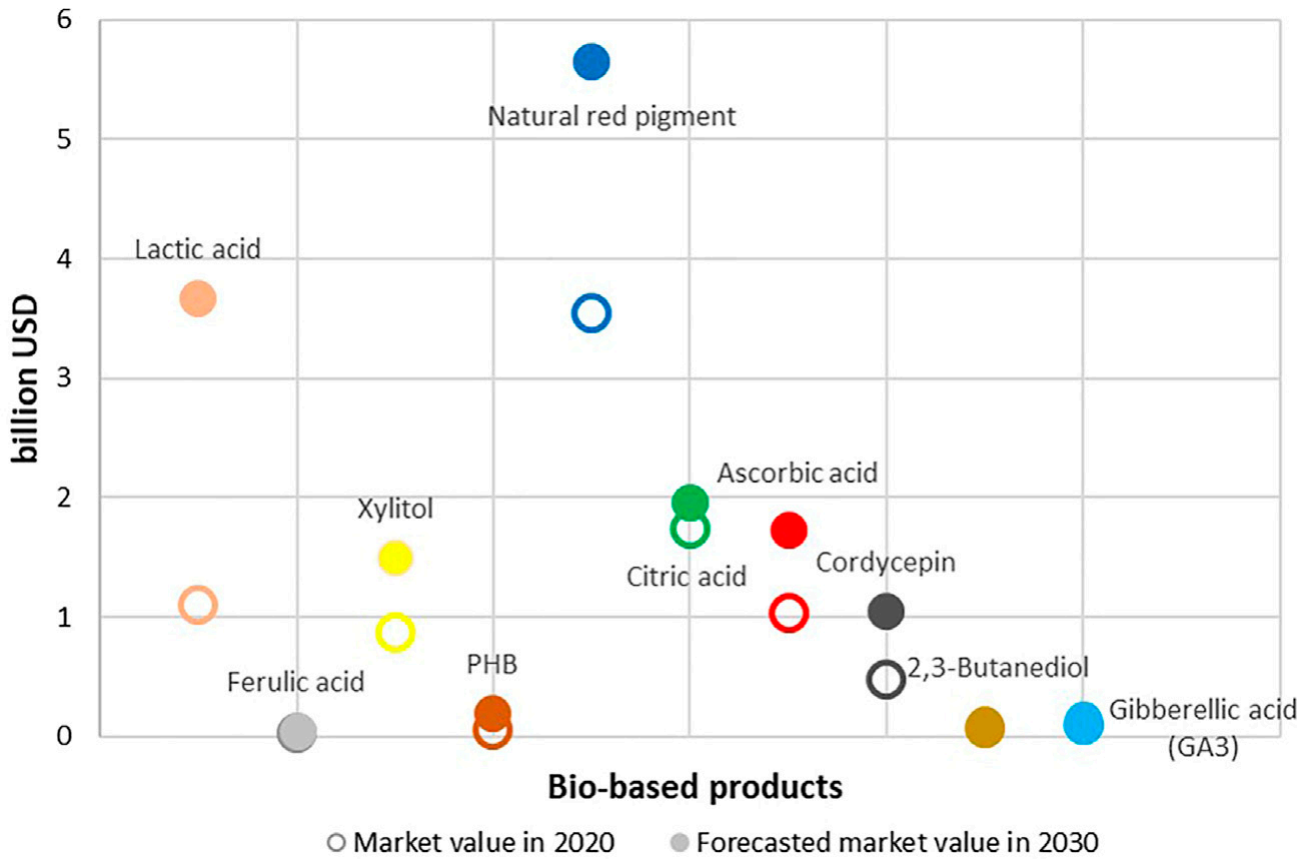
Market shares of high-value BSG-based products (PHB as a part of the PHA group; Natural red pigment as a part of natural pigment group) in 2020 and estimation for 2030.

**FIGURE 3:**
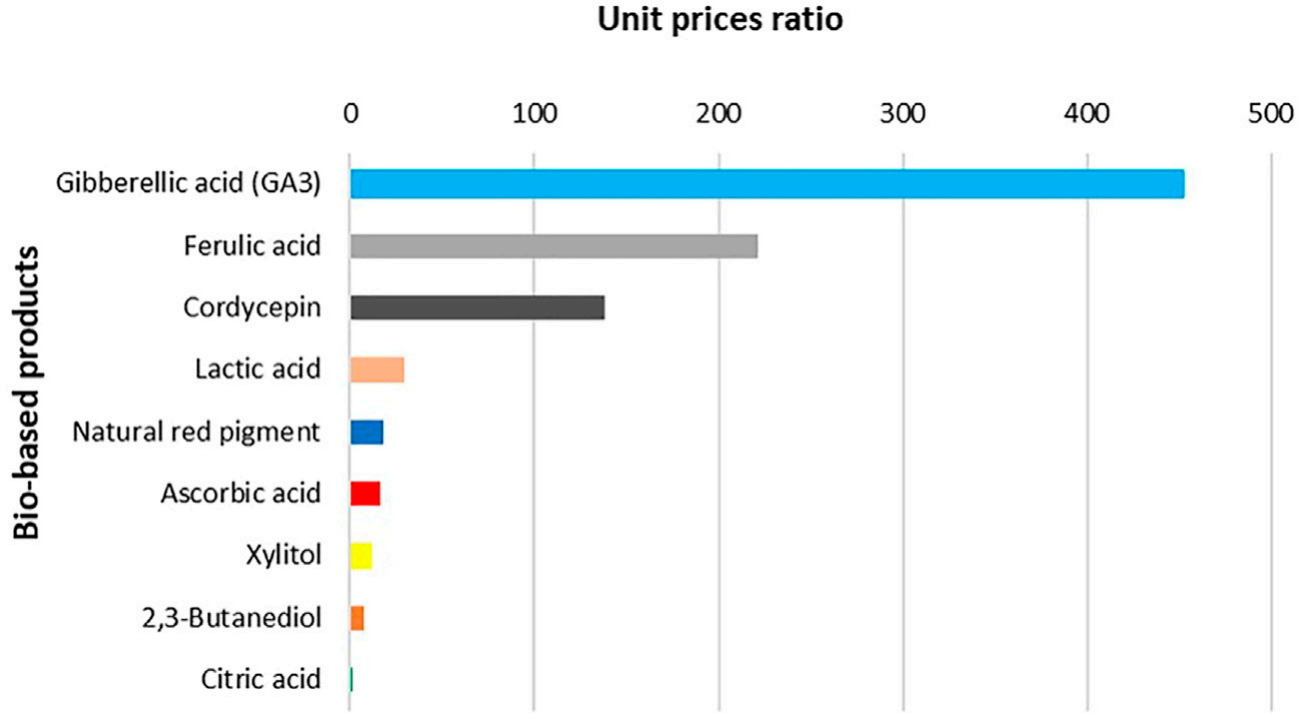
Relative ratios of unit prices per selected value-added products from BSG.

While BSG has numerous promising pathways from waste to high value added product, it is useful to contrast the research potential with the potential market size where such novel product will be placed. Another valuable information provides the approximate unit price of the novel BSG product to size if the effort invested could be covered with the unit price achieved. While the lab scale work is much different than the industrial production, a researcher can navigate through market strata with the achieved yield of the desired component per BSG unit (Table 4), lab costs and approximated unit market price. BSG provides numerous opportunities to increase resource efficiency and sustainability, yet those opportunities seldom stay within the original, beer production. Sizing market opportunities to increase national resource efficiency relies much on recognising and forming strategic partnerships described in circular business models (Salvador et al., 2021). When deciding on the circularity pathway of BSG in the emerging circular and sustainable bioeconomies, matching available intellectual capital and BSG availability with bio-based alternatives to fossil components demand, it is worth examining the market potentials. Figure 1 illustrates the relative sizes of selected potential products of BSG valorization in world markets, and the corresponding compound annual growth rate (CAGR). The idea behind Figures 1–3 is to size and compare different potential markets for novel products as well as to align unit prices to recognise and form strategic partnerships between the BSG owner (i.e., brewery) and novel BSG-based product demand industry, whether to form market penetration strategy or to develop a circular business model.

Market size data presented in Figure 2 of selected bio-based products is retrieved from numerous websites that report on market shares and forecast future values based on the CAGR without preferring any of the sources, such as: Markets and Markets (Lactic acid); Market Watch (Ferulic acid; 2,3—Butanediol; Gibberellic acid); Expert Market Research (Xylitol, Citric acid), Business Wire (PHB), Market Research Future (Natural Red Pigment), Reports and Data (Ascorbic acid), Grand View Research (Cordycepin).

The highest annual growth rate (Figure 1) is forecast for lactic acid, which is also expected to be the largest single market for BSG-based products in 2030 (Figure 2). Natural red pigment is shown as a part of the natural pigments group, which has a large existing market worth 3.5 billion USD and a projected CAGR of 4.75% by 2025. Other uses of BSG are in a range of 1-2 billion USD, with the exception of ferulic acid and PHB.

However, when looked at the unit price (derived by triangulation of an average wholesale price at Alibaba.com) ratio, Gibberellic acid is by far carrying the greatest unit value per kg. Figure 3 illustrates ratios between unit prices among the highly valued bio-based products from BSG.

Considering the current linear structure of bioeconomies in the BIOEAST macro-region (Kulisic et al., 2020), the use of lactic acid from BSG in the existing food and beverage, pharmaceutical and cosmetics industries, as well as for biopolymers production allows a rapid transition to bio-based industry by giving BSG circularity in the macro-region. Most effects from strategic partnerships with brewery industry in lactic acid, but not limited to it, are expected in countries with high beer production and potential demand from the industry to replace fossil carbon with renewable one, supplied by short supply chains: Poland, Romania, Czech Republic, followed by Bulgaria, Croatia, Lithuania and Slovakia. In Bulgaria, Croatia, Lithuania and Romania, beer production belongs to the top 10 industrial products with their contributions to the gross value added. In those countries, circularity of BSG might fortify the competitiveness of the beer industry.

## Conclusion and Future Research Directions

Although there are a large number of publications dealing with the application of BSG for the production of biofuels (biogas, bioethanol, biobutanol), biofertilizers, value-added products such as enzymes (mainly xylanase), lactic acid, ascorbic acid, citric acid, gibberellic acid, ferulic acid, xylitol, etc.), its use in biorefinery is still rare.

Future directions should aim to *a*) develop rapid and accurate methods to determine the chemical composition of BSG, *b*) cascade utilization of BSG based on its composition to produce value-added products and biofuels, *c*) develop sustainable methods for pretreatment of BSG, fractionation of polymers, and isolation of value-added products, *d*) implement methods to assess sustainability of all process phases involved in BSG utilization within biorefinery to follow the concept of circular and sustainable bioeconomy.

In this way, keeping BSG in the production cycle by producing value-added biobased products could be a readily available option for the industry to transition to a sustainable and circular bioeconomy, recognize, expand and define their future strategic partnerships with the brewing industry.

## Footnotes

1
https://www.bbi.europa.eu/search/node/lignocellulosic%20type%3Aprojects%20%22food.

2
https://bioeast.eu/home/

3
https://www.statista.com/statistics/202417/beer-output-volumes-of-the-different-continents-in-2010/

4
https://brewersofeurope.org/site/countries/key-facts-figures.php
