# Supplementary material for: The Potential of Brewer’s Spent Grain in the Circular Bioeconomy: State of the Art and Future Perspectives
Source: Front Bioeng Biotechnol. 2022 Jun 17;10:870744. doi: 10.3389/fbioe.2022.870744 (PMC9247607; doi:10.3389/fbioe.2022.870744)
Supplement: Supplementary file 1 [file Table1.docx]

Supplementary Table 1. Overview of BSG pretreatment methods for its further use in various applications (energy production, biofertilizer production, waste management, feed and food production)

| Target group | BSG pretreatment method and fermentation* | The aim | Efficiency | Literature |
| --- | --- | --- | --- | --- |
| ENERGY PRODUCTION | | | | |
| Bioethanol | P1: Alkaline pretreatment (NaOH or H_2_O_2_)  P2: Enzymatic hydrolysis (cellulolytic enzymes)  F: by *S. cerevisiae* | Effect of alkaline pretreatment and enzymatic hydrolysis for sugar release, lignin degradation and bioethanol production. | Mean ethanol concentrations of 17.3 g/L and 8.4 g/L were achieved for the NaOH and H_2_O_2_ fermentations, respectively, which corresponded to 81% and 43% of theoretical ethanol yields. | Wilkinson et al. (2014) |
|  | P1: Acid hydrolysis (H_2_SO_4_)  P2: Alkaline hydrolysis (NaOH)  F: by *S. cerevisiae* | Effect of hydrolysis and potential of BSG on bioethanol production and yield. | The ethanol content in the acid-treated BSG was 1.90%, while the value of ethanol for alkaline treated BSG was not significant. | Olugbenga and Ibiyemi (2011) |
|  | P1: Thermoplastic extrusion  P2: Enzymatic hydrolysis (exoglucanase, endoglucanase, β‑glucosidase, hemicellulase and xylanase)  F: by *S. cerevisiae* | Effect of extrusion conditions and hydrolysis, with fiber degrading enzymes, on the production of C5 and C6 sugars from BSG for bioethanol production. | The ethanol yield was 5.43 mL/100 g BSG. The amount of free amino nitrogen quantified in the hydrolyzate negatively affects sugars consuption during fermentation and ethanol yield. | Heredia-Olea et al. (2015) |
|  | P1: Acid hydrolysis (HCl and HNO_3_)  P2: Enzymatic hydrolysis  F: by *Pichia stipitis* and *Kluyveromyces marxianus* | Optimization of sugar fermentation from BSG to bioethanol, based on pentoses. | The theoretical ethanol yield from BSG hydrolyzates was 0.27 and 0.19 g ethanol/g of sugars, *P. stipitis* and *K. marxianus*, but the ethanol yield obtained is 0.0856 and 0.0308 g ethanol/g of sugars. | Mata et al. (2015) |
| Biobutanol | P1: Laccase preatreatment  P2: Enzymatic hydrolysis (cellulases, xylanase and amylase)  F: by *Clostridium acetobutylicum* | Ability of two laccase preparations from *Pleurotus ostreatus* to degrade lignin and detoxify ground BSG. The purpose is to improve saccharification and butanol production by Acetone-Buthanol-Ethanol (ABE) fermentation. | *P. ostreatus* laccases successfully detoxified and delignified BSG, 94% of phenolic compounds, which are inhibitors of ABE fermentation, were removed. Enzyme pretreatment avoided sugar degradation. 3.8 g/L of acetone, 7.8 g/L of butanol and 1.0 g/L of ethanol were produced. | Giacobbe et al. (2019) |
|  | P1: Acid hydrolysis (1 M H_2_SO_4,_ 5% and 15% BSG)  P2: Enzymatic hydrolysis (cellulases and β-glucosidases)  F: by *Clostridium beijerinckii* DSM 6422 | Effect of acid treatment and enzymatic hydrolysis separately on the production of butanol from BSG with ABE fermentation. The benefits of washing BSG before saccharification and detoxification were investigated. | Pretreatment at 15% BSG resulted in higher availability of sugars in both pretreatment and overall yields of 75 g butanol/kg BSG and 95 g ABE/kg BSG were obtained. When the enzymatic hydrolysate from the washed pretreated BSG was fermented, butanol (6.0 ± 0.5 g/L) and ABE (7.4 ± 1.0 g/L) concentrations were lower compared with 7.5 ± 0.6 g/L butanol and 10.0 ± 0.8 g/L ABE from a control. | Plaza et al. (2017) |
| Pyrolysis biofuels | P1: Soaking in a 0.1 M aqueous silver nitrate (AgNO_3_) solution  P2: Thermal treatment (*T* = 500 °C) | Use of BSG for in situ catalytic upgrade of pyrolysis fuel, by impregnation of AgNO_3_ into BSG to reduce pyrolysis temperature and improve process economy. | Ten times higher increase in hydrogen gas evolution, with a 64% increase in ethane production in the impregnation of BSG with silver nitrate, compared to the conventional way. | Ashman et al. (2020) |
| Biogas | P: Thermo-chemical hydrolysis (HCl, *T* = 95 °C, *t* = 4 h) | Potential of biogas production from BSG using two stages of anaerobic digestion. | 120 L biogas/kg wet BSG was achieved. The two-stage process proved to be better in terms of yield, biodegradability and methane yield. | Panjičko et al. (2017) |
| BIOFERTILIZER | | | | |
| Biocontrol fertilizer | F: SSF by fungi *Beauveria bassiana* (BbQLU1) | BSG as substrate in fermentation for biocontrol fertilizer production. Fermentation time, optimal water content and temperature influence were investigated. | The optimum conditions for BF production (60% water content at 25 °C for 12 days) resulted in a conidiation of 0.85 × 10^8^ spores/g and germination rate of 98.68%. BF at a concentration of 1 × 10^−2^ g/mL prompted plant growth and exhibited high toxicity. GC-MS analysis found 2-piperidone; benzoic acid, 3-methyl-, methyl ester; and other compounds to be potentially related to the toxicity and enhanced plant growth. | Qiu et al. (2019) |
| Vermicompost | P1: Thermostating with the addition of sterile distilled water (*T* = 22 °C, *t* = 17 days)  P2: Vermicomposting (BSG, cow manure and *Eisenia fetida*) | Potential of low-input treated BSG as vermicompost and assessment of microbiological and chemical analysis of the process. | BSG microbiota was enriched in bacterial and fungal species including lactic acid bacteria (*Weissella, Pediococcus*), plant growth-promoting bacteria (*Bacillus, Pseudomonas, Pseudoxhantomonas*), and biostimulant yeasts (*Pichia fermentans, Trichoderma reesei, Beauveria bassiana*). The C/N ratio was 11, organic carbon 38.63 g/kg and total nitrogen 32.8 g/kg | Bianco et al. (2022) |
| WASTE MANAGEMENT | | | | |
| Ag (I) removal | P: Modification of spent grain with 1 M NaCl solution | The capability of raw spent grain (RSG) and modified spent grain (MSG) to adsorb Ag (I) from waste aqueous solution was investigated and compared with raw spent grain (RSG) regarding their adsorption isotherms, kinetics, and thermodynamics. | The Ag (I) adsorption on MSG was 4 times higher than that on RSG. The monolayer adsorption capacity was 30.28 mg/g for RSG and 158.23 mg/g for MSG according to the Langmuir isotherm. | Li et al. (2010) |
| BIOFILMS | | | | |
| Thermoplastic biofilms | P1: Alkali extraction (ethanol, *T* = 25 °C, *t* = 8 h)  P2: Acid extraction (3 M HCl)  P3: Freeze-drying | Production of biofilms from the arabinoxylan-rich fraction from BSG by addition of two plasticizers (glycerol and polypropylene glycol) and a anti-foaming agent (coconut oil). | The value of the Zeta potential was -3.44 to 9.17 mV. Two degrees of weight loss have been observed in plasticized films. The addition of plasticizers and anti-foaming agents increases the Ea of biofilms. Biofilms have shown microbiological safety. | Jaguey-Hernández et al. (2022) |
| Protein  biofilms | P1: Alkali extraction (alkaline solubilization), *T* = 25 °C, *t* = 2 h)  P3: Spray drying (inlet *T* = 170-190 °C, outlet *T* = 80-90 °C) | Production of protein films (BSG-PC) from BSG with potential application in active packaging. Protein dispersions were prepared at different pH (2, 8, 11) using two plasticizers, polyethylene glycol (PEG) and glycerol in the range of 0-0.25 g/g of PEG. | The formulation with 0.10 and 1.15 g PEG per g BSG-PC and pH 2 shows balanced mechanical properties, waterproofing properties and antioxidant capacity. The addition of 0.10 g PEG /g BSG-PC increases the structure of α-helix, due to higher tensile strength and modulus of elasticity of the films. | Proaño et al. (2020) |
| FEED PRODUCTION | | | | |
| Chicken feed | P: Enzyme treatment whit liquid xylanase (activity = 7250 GXU/g, concentration of 2500 GXU/kg, modified mixer, *t* = 3 h, *T* = 50-55 °C) | Feeding broilers with BSG of different particle sizes (fine and coarsely ground) pretreated with xylanase. The effect of xylanase on the performance and concentration of neutral sugars (arabinose and xylose) was investigated. | Xylanase pretreatment reduced the reduced the concentration of polymeric arabinose and xylose by 15–30%. Feed utilisation was significantly higher (6%) when the birds were given coarse BSG. The overall starch digestibility was high (99%), with no dietary differences, whereas ileal protein digestibility was low (57%). | Denstadli et al. (2010) |
| Fish feed | P: SSF by fungi *Aspergillus ibericus* | BSG extract rich in carbohydrates after SSF was included in the plant diet of fish in the range of 0.1-0.4%. Digestibility tests, dietary phosphorus, phytate phosphorus, carbohydrates, protein hydrolysis, and enzyme interactions were evaluated. | The inclusion of 0.4% BSG extract in the plant diet for fish increases the hydrolysis of phosphorus and carbohydrates. Endogenous fish enzymes reduce the effectiveness of BSG extract by about 7% to 16%. There was an increase in the digestibility of dry matter, energy, starch, cellulose and glucan. | Fernandes et al. (2021) |
| FOOD PRODUCTION | | | | |
| Functional food ingredient (Erinacine C-enriched *Hericium erinaceus*) | P: Acid hydrolysis (0.2 M H_2_SO_4_)  F: SmF by *Hericium erinaceus* | BSG as nutrient media (after being pretreattted with acid) for SmF cultivation of *H. erinaceus* for the purpose of erinacene C. | Acid pretreatment resulted in a bioconversion of 38.6%. The highest cell dry weight was obtained on day 21 yielding 7.14 g/L and concentration of erinacine C metabolite was 174.8 mg/g. | Wolters et al. (2016) |
| Whole-wheat bread | F: SSF by *Aspergillus awamori* in tray-bioreactor (*T* = 30 °C, air water saturation = 90%, *t* = 96 h, 55% (w/w)) | Properties of bread produced with the addition of BSG which was previously treated with *A. awamori* IOC-3914, under solid-state condition aluate their addition in breads. | Bioprocessing breads with fermented BSG led to an increase in soluble ferulic acid of 198%, without impairing its technological properties. | Dos Santos Costa et al. (2020) |

**P – pretreatment; P1 – 1^st^ step of pretreatment; P2 – 2^nd^ step of pretreatment; F – fermentation.*
